# The age-phenome database

**DOI:** 10.1186/2193-1801-1-4

**Published:** 2012-04-23

**Authors:** Nophar Geifman, Eitan Rubin

**Affiliations:** 1 Shraga Segal Department of Microbiology and Immunology, Faculty of Health Sciences and The National Institute for Biotechnology in the Negev, Ben Gurion University, Beersheva 84105, Israel

**Keywords:** Age, Phenotype, Knowledgebase, Text-minig

## Abstract

Data linking specific ages or age ranges with disease are abundant in biomedical literature. However, these data are organized such that searching for age-phenotype relationships is difficult. Recently, we described the Age-Phenome Knowledge-base (APK), a computational platform for storage and retrieval of information concerning age-related phenotypic patterns. Here, we report that data derived from over 1.5 million human-related PubMed abstracts have been added to APK. Using a text-mining pipeline, 35,683 entries which describe relationships between age and phenotype (such as disease) have been introduced into the database. Comparing the results to those obtained by a human reader reveals that the overall accuracy of these entries is estimated to exceed 80%. The usefulness of these data for obtaining new insight regarding age-disease relationships is demonstrated using clustering analysis, which is shown to capture obvious, as well as potentially interesting relationships between diseases. In addition, a new tool for browsing and searching the APK database is presented. We thus present a unique resource and a new framework for studying age-disease relationships and other phenotypic processes.

## Background

The relationship between age and human health has been extensively investigated over the years. Such studies have identified a plethora of so-called age-related diseases (Wick et al. 2000). A patient's age may effect the course and progression of a disease (Diamond et al. 1989, Hasenclever and Diehl 1998) or may be an important factor in determining the correct course of treatment (Vecht 1993). As a result of these investigations, a significant quantity of data exists linking specific ages or age ranges with disease, as well as with other clinical phenotypes, such as 'normal' parameter values from blood tests.

We have previously described the Age-Phenome Knowledge-base (APK) in which knowledge about age-related phenotypic patterns and events can be modelled and stored for retrieval (Geifman and Rubin 2011). The knowledge-base holds a structured representation of knowledge, derived from scientific literature and clinical data, about clinically-relevant traits and trends which occur at different ages, such as disease symptoms and propensity. Disease and age are described using ontologies, allowing for abstraction in searches (for example, searching for evidence linking "infectious diseases" and "children" instead of searching for a specified list of diseases and a range of ages). In the APK, ages and phenotypes are linked via textual snippets that describe the connections between them. Furthermore, the type of connection between age and phenotype is described using one of five pre-defined relationships (i.e. age of onset, age of diagnosis, age of observation, age of occurrence and age of evaluation).

Biomedical text is rich in age-dependent trends and description of relationships between age and phenotype. These are freely available in the form of PubMed (2011) abstracts. Information about age-related trends may thus be obtained by mining the pertinent information contained in this resource. Numerous efforts to extract information from PubMed using text-mining tools have been described (Donaldson et al. 2003, Temkin and Gilder 2003, Chen and Sharp 2004). However, these efforts usually focus on the extraction of biological information, such as relationships between genes, RNA and proteins (Hirschman et al. 2002, Shatkay and Feldman 2003). Only few approaches for extracting medical and clinical information from biomedical abstracts are available. These include tools, such as EDGAR (Rindflesch et al. 2000), which extracts information about drugs and genes relevant to cancer from the biomedical literature, and MetaMap (Aronson 2001), which is a highly configurable program that maps biomedical texts to concepts in the Metathesaurus of the Unified Medical Language System (UMLS) (Bodenreider 2004). The Metathesaurus is a large vocabulary database that contains information about biomedical and health-related concepts, their various names, and their inter-relationships. Unfortunately, while MetaMap can be used to map biomedical abstracts to the UMLS, initial testing suggested that applying it to over a million PubMed abstracts will be computationally impracticable.

Other text-mining tools and techniques have been specifically developed for the extraction of medical and clinical information from clinical (patient) records (Zou et al. 2003, Zhou et al. 2006, Voorham and Denig 2007, Uzuner et al. 2008). One such tool is MedLEE (Friedman et al. 1995), which extracts, structures, and encodes clinical information from textual patient reports. Methodological testing and evaluation have demonstrated the applicability of MedLEE to several clinical fields, ranging from chest radiographs to pathology reports (Friedman et al. 1994, Xu and Friedman 2003). Though MedLEE may be very useful in information extraction from clinical records, it was not designed for the purpose of mining PubMed abstracts.

While in the past we have described the knowledge model of the APK, populating it with only a sample set of data, here we present the analysis of over 1.5 million human-related abstracts from PubMed (2011), using a simple text-mining pipeline developed for this purpose. The pipeline, which was designed to emphasize accuracy over sensitivity, achieves an estimated overall accuracy of ~82%. The resulting database contains 35,683 entries which describe relationships between age and disease. We present a new tool for browsing and searching the APK database and demonstrate how the data stored in the APK can be used to generate hypotheses regarding the connection between age and phenotypes such as disease.

## Results

### Knowledge extraction and quality

We have developed a knowledge extraction pipeline which was used to mine all human-related PubMed abstracts published since 1990 (over 1.5 million abstracts). For each of the 51,966 abstracts deemed relevant to age, the mentioned age was extracted and one of five predefined age-phenotype relationship was assigned. A relationship was successfully identified for approximately 50% of the abstracts, with an estimated accuracy of 88% (see below). Textual snippets generated from these abstracts where mapped to terms from the Disease Ontology (DO) and a subset of UMLS concepts using a simple lexicon-based method. Mapping to the DO and the UMLS was performed by the Mapper.pl tool we developed for this purpose (see Materials and Methods).

**Figure 1:**
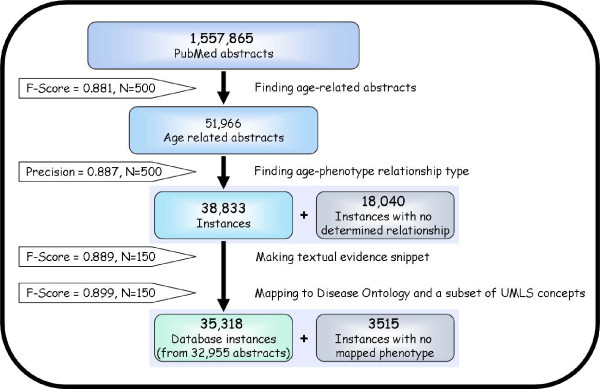
**Data-mining pipeline**. The process of mining PubMed abstracts can be divided into 4 main steps: 1) Finding age-related abstracts, 2) determining the age-phenotype relationship type for those age-related abstracts, 3) generating a textual snippet which describes the most important information given in the abstract regarding the captured age and 4) mapping the text snippets to phenotypes from the DO and a subset of the UMLS. Instances for which an age-phenotype relationship could not be determined or no phenotype were found were not used to populate the database.

Each of the 4 main steps of the knowledge-mining process was evaluated by comparing the text mining results to those obtained by a human reader (Figure 1), similarly to (Hripcsak et al. 1995, Friedman and Hripcsak 1998, Meystre and Haug 2006, Zeng et al. 2006). Performance quality of text-mining and information extraction techniques is generally measured in terms of precision (the fraction of retrieved instances that are relevant) and sensitivity (the fraction of relevant instances that are retrieved).

1. *Finding age-related abstracts*. On this task, our tool performed with a sensitivity of 0.7867 and precision of 1 (F-score = 0.881), as estimated from 500 abstracts. We found no errors in the age that was extracted from age-related abstracts, though for some instances, a more accurate age range could have been extracted. For example, even when a precise full range of ages was mentioned in one part of the text, the tool extracted the average age of the studied group of patients which was mentioned in another part. In total, 92.4% of the age ranges were extracted perfectly.

2. *Determining a pre-defined age-phenotype relationship type*. For 49% of the entries, Miner.pl assigned a correct relationship type and for 44.8% of cases, Miner.pl could not determine the relationship type. Miner.pl incorrectly identified the relationship type for only 11.2% of the abstracts (for which a relationship was assigned). It should be noted that in the Age-Phenome database, only instances with a determined relationship are included. Therefore, based on the evaluation results, for approximately 88.75% of the instances stored in our database, the relationship assignment is correct.

3. *Generating a textual snippet*. Miner.pl performed on this task with a precision of 0.968 and sensitivity of 0.822 (F-score = 0.889); 80% of the textual snippets generated by Miner.pl contained all of the most important information, as compared to the snippets generated by the human reader. 2.7% of the snippets generated by Miner.pl contained unrelated (erroneous) information and 17.3% where missing important information regarding the captured age. For the majority of the mined abstracts only one database instance was generated. For only 2024 of the abstracts which were used to populate the database 2 or more instances were generated.

4. *Mapping to the Disease Ontology and a subset of the UMLS*. Mapped phenotypes where considered to be true positives or false positive. Phenotypes missed by Mapper.pl where considered as false negatives, while missed phenotypes which where not included in the DO or the subset of the UMLS where considered as true negatives. Some phenotypes which were mapped to instances by Mapper.pl were considered as harmless. These 'harmless' mappings may add redundant mapping or mapping to less important phenotypes but do not add erroneous mapping. For example, in the text *"A 59-year-old white man was diagnosed with primary squamous cell carcinoma at the base of the tongue*", the disease '*squamous cell carcinoma*' is the main phenotype this instance was mapped to; in addition, it was mapped to the term *'carcinoma' *, which only adds harmless redundancy. When these harmless added phenotypes were not including in the calculation, Mapper.pl performed with a precision of 0.93 and sensitivity of 0.869 (F-score = 0.899). If the phenotypes considered as harmless are deemed as false positives, the F-score for this task is 0.628. We further compared Mapper.pl performance in this task to TermFinder, a previously published tool (Teufel and Elhadad 2002) which maps text to concepts from the UMLS. TermFinder was applied to the same 150 instances and the results were compared to the human annotator's results. TermFinder performed with an F-score of 0.922 (or 0.602 if harmless mapping is considered as false positives), which is only better than Mapper.pl by 2.5%. We thus chose to use Mapper.pl rather than using TermFinder, as Mapper.pl directly links to the DO, while TermFinder requires an additional step of mapping from the UMLS to the DO, a step which could introduce additional errors.

### The APK database

**Figure 2:**
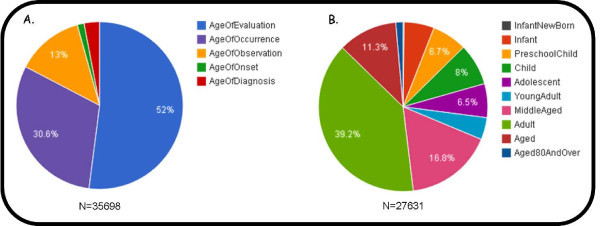
**Database contents summary**. A. Instances per relationship type. B. Instances per Age Ontology age class. An instance is assigned the most specific Age Ontology class which contains the whole of the age range to which that instance is linked. The age ranges of each class are as follows: Infant new born: 0-1 month; Infant: 0-2 years; Child: 2-12 years; Preschool child: 2-6 years; Adolescent: 12-18 years; Adult: 18-120 years; Young adult: 18-24 years; Middle aged: 45-64 years; Aged: 64-120 years; 80 years or over: 80-120.

As discussed above (Figure 1), mining of all PubMed abstracts yielded over 35,000 instances which were used to populate the APK database. Furthermore, the database also contains 239 previously described instances of links between age and phenotype which were extracted from clinical data obtained from the Third National Health and Nutrition Examination Survey. A summary of some of the database content is provided in Figure 2.

**Figure 3:**
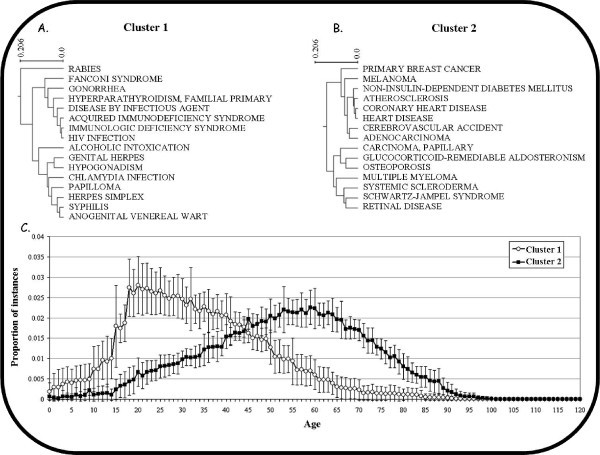
**Hierarchical clustering analysis results: Two examples**. A) A phylogram of cluster no. 1, in which many sexually-transmitted diseases (STDs) are clustered. B) A phylogram of cluster no. 2, in which several types of cancer and cardio-vascular diseases are clustered. C) Graphical representation of clusters 1 and 2. For each cluster, the average number of instances per age per disease was plotted. For cluster 1, literature reports peak in the late teens - early 20s, while for cluster 2, literature reports peak at around age 60.

To evaluate the ability of the knowledge stored in the APK database to correctly represent and summarize current knowledge regarding age in health, we conducted clustering analysis of the data (see the Methods section for details), grouping together diseases that are associated with similar ages. Hierarchal clustering of disease by age revealed several clusters that clearly mirror medical knowledge of disease-age relationships (Figure 3). This type of analysis may be used to generate new hypotheses regarding links between diseases which, according to biomedical literature, appear at the same ages. However, such an analysis is beyond the scope of this paper since it requires the clustering process to be optimized.

### The age-phenome wiki

We have previously described the Age-Phenome Wiki, an interface which was developed as a means to share the knowledge stored in the APK and to harness the knowledge of the user community for further enriching the knowledge in APK (Geifman and Rubin 2011). The knowledge shared via this wiki has been updated but due to the large amount of data now available in the APK, only a subset of the knowledge is made available using this platform. We have selected to display only evidence instances which link age and phenotype with the 'Age of onset', 'Age of diagnosis' and 'Age of observation' relationships, thus excluding evidence linked via the 'Age of occurrence' and 'Age of evaluation' relationships. Furthermore, evidence linked to an inferred age range (such as in cases where the text mentioned "30 years or less", when the instance was linked to ages 0-30) were excluded. With these limitations in mind, the APK Wiki is available for use. Changes are instantaneously made available to the editable Wiki, and are typically introduced to the readable wiki within a few working days.

### The APK data browser

**Figure 4:**
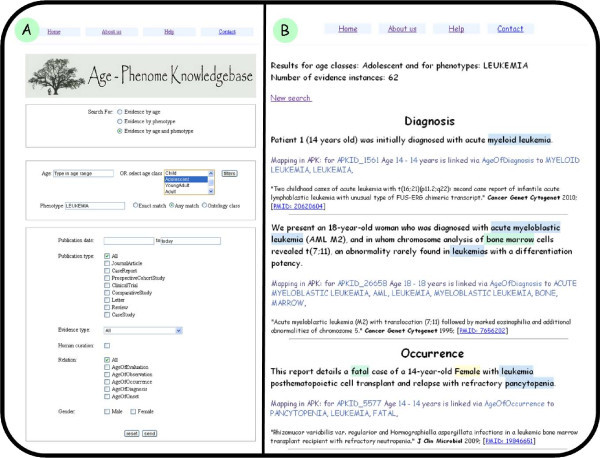
**The APK data browser**. A) The APK data browser search form. In this example, the selected search type is by age and phenotype, the 'Adolescent' age class is selected and the phenotype used for this query is 'Leukemia'. B) Results for the search for evidence linked to the age class 'Adolescent' and the disease 'Leukemia'. Results are displayed according to the type of the assigned age-phenotype relationship.

Users who are proficient with SQL can query the APK using a formal query language. However, for the computationally naïve user, a user-friendly query engine was developed. Three types of queries can be performed with the search engine: 1) Search for evidence by age, 2) search for evidence by phenotype and 3) search for evidence by age and phenotype. Queries can be refined by restricting the publication year, publication type (e.g. research article, review, etc.), human curation status, relationship type and gender. Therefore, the data browser makes it possible for users to answer questions, such as what diseases are linked to an age or age-range of interest or what ages or age groups are linked to a phenotype of interest. Queries can be made more complex, for example by searching for evidence linking ages to more than one phenotype or searching for evidence linked to both a phenotype and age of interest. Query results are displayed as html in a fashion very similar to that employed by the APK wiki (Figure 4).

An interesting feature of the data browser results from the use of the DO, a formal ontology, to represent diseases. Unlike simple keywords, the ontology structures provides hierarchical abstraction, searching for high level terms works, like searching for cancer, for example, will provide all the instances that involves any type of cancer (and which are specified in the DO).

## Discussion

We present here the APK database and discuss its population with knowledge extracted from over 1.5 million PubMed abstracts. The performance of the pipeline used to generate APK was assessed by comparing the results to user curation, and each step was shown to achieve high accuracy and sensitivity. While text mining techniques are very useful in extracting information from large corpuses of texts, they are still somewhat limited. In mining abstracts for age-phenotype information, these limitations include missing information such as phenotypes of importance to the mentioned age or adding incorrect information such as the wrong phenotypes or relationship. Furthermore, our text mining tool was not designed to capture conditional relationships. For example, an abstract may describe the age in which ulcers are observed in HIV-positive patients. Our current text-mining tools would create a link between the age range specified in the abstract and HIV as well as ulcers, rather than capture the conditional relationship between the phenotypes and age. As expected, our pipeline did not capture all of the age-related information available in biomedical abstracts. Yet, with only few errors of commission, the data generated is of good accuracy.

We are aware that the evaluation approach we used for mapping to phenotype is biased toward detecting erroneous information and that errors of omission can be missed with this approach. However, since a large body of information was captured, and since no other knowledge-base of age and phenotypes currently exists, we consider accuracy to be more important than completeness. The APK can now serve as a baseline for future efforts to improve the completeness, as well as the accuracy of age-phenotype extraction from PubMed.

Using hierarchal clustering, we show that knowledge stored in the APK can be used to capture the current medical knowledge in at least two examples. In the first example (Figure 3a), sexually transmitted diseases (such as HIV, Gonorrhea, Herpes and Chlamydia) are clustered by their links to age. Furthermore, the distribution of instances linked to diseases in this cluster peaks in the late teens-early 20s, in agreement with current views of STDs and youth (Weinstock et al. 2004). The other cluster (Figure 3b) captures the fact that some cancer types and cardiovascular diseases are age-related by linking several such diseases and associating them with a peak of instance distribution at around age 60.

These analytic techniques, along with others, can be used to analyze the data in the APK and possibly generate new hypotheses regarding connections between phenotype and age. For example, consider an analysis that shows that disease A is repeatedly mentioned in the literature as occurring at the same ages as does disease B. It can be hypothesised that since these two disease share the same pattern of literature reports, they may also share a medical or biological mechanism. Though such hypotheses would need to be further investigated (for example, to rule out the possibility of reporting biases), we believe that the APK provides its users with the opportunity to generate many valid hypotheses.

## Conclusions

We present here an extensive database of age-phenotype connections extracted from biomedical abstracts, with the tools required to mine and improve such predictions. We believe that this database, along with the user interfaces provided by the Age-Phenome Knowledge-base, will become a great asset to the biomedical research community.

## Methods

### Text mining

For a complete description of the text mining process, see online supplementary files at: http://rubinlab.med.ad.bgu.ac.il/apk/APKSupplementary.html. Briefly, PubMed abstracts were exported from the NCBI in the MEDLINE format, using the key word 'human'. The resulting text files where mined using two processing scripts:

1. Miner.pl - This script uses regular expressions to determine if an abstract mentions a specific age or age range, retrieves the mentioned age or age range and determines which of the five pre-defined relationships (age of onset, age of diagnosis, age of observation, age of occurrence and age of evaluation) should be assigned to the abstract. In cases where an age range is not fully constrained (e.g. " 30 years or less"), the instance is linked to all the possible ages covered by the expression, and the information indicating that the full range was inferred is saved. Note that the current set of regular expression only looks for ages that are described numerically; textual references to age (e.g. "Two to five years old") were not parsed.

A second set of regular expressions is used to determine the type of age-phenotype relationship described in the abstract. We note a successful assignment of an age-phenotype relationship is required; this implies that the text mining process goes beyond co-occurrence of keywords in the same abstract.

Finally, a text snippet is generated (usually 1-3 sentences) that best captures the most important phenotype/health-related information related to the captured age. The snippet is relatively short and may be constructed of non-consecutive sentences (though sentences are always kept whole). The process of selecting sentences to construct the evidence text snippet is based on the type of relationship assigned to the captured age, whether the abstract is structured, existence of a concluding sentence, whether there are sentences talking about patients or subjects, etc. A full description is provided in the online supplementary file, 'Make snippet'.

2. Mapper.pl - This script maps text snippets (generated by Miner.pl) to diseases and other phenotypes. A list of disease names and synonyms that are described in the Disease Ontology are sought in the text using perfect-match searching. After mapping to the Disease Ontology, a selected list of concept names and synonyms from the UMLS are also sought in the text. The subset of UMLS concepts used here includes diseases, drugs and findings (a full list of the types of concepts included in the subset is provided with the Miner.pl script). If a UMLS concept is found in the text that is defined as a disease, the script maps the text snippet to the DO disease linked to that UMLS concept (based on mapping between the DO and the UMLS provided in the DO). For other types of concepts (not diseases), both the concept name and unique concept ID are saved. In addition to mapping each text snippet to the Disease Ontology and to a subset of concepts from the UMLS, the script also determines whether gender is mentioned. Gender is assigned to an evidence instance if only one gender is mentioned in the text snippet.

### Data quality evaluation

Evaluation of the quality of the data captured from PubMed abstracts and stored in the Age-Phenome Database was performed by comparing the results of the knowledge-mining process to those achieved by a human reader. The performance of each of the four knowledge-mining steps was assessed as follows:

1. *Finding age-related abstracts*. To evaluate the performance of Miner.pl in this task, five-hundred PubMed abstracts where randomly selected (100 for each of the following diseases: Bipolar disorder, Schizophrenia, obesity, Parkinson's disease and Glioblastoma, chosen for their diversity and familiarity to our human evaluator). All abstracts where read by a human evaluator who decided whether an abstract included age-related information. Abstracts where considered to be age-related only if a specific age was mentioned (e.g. age 30). The results obtained by a human reader where compared to those obtained by miner.pl.

2. *Determining an age-phenotype relationship type*. To evaluate the performance of Miner.pl at this task, the human reader decided which of the five pre-defined relationship best suits the age captured for each of the abstracts deemed as age-related (out of the 500 used to evaluate task 1). The results where compared to those obtained by Miner.pl.

3. *Extracting a textual snippet from an abstract*. For the evaluation of this task, 150 instances in the database where randomly selected. For each instance, the full abstract was used to generate a textual snippet by a human reader. The human-generated snippets were then compared to those generated by Miner.pl. Text snippets generated by Miner.pl where regarded as containing all of the important information regarding the captured age, missing important information or containing unrelated information (i.e. erroneous), in comparison to those generated by the human reader.

4. *Mapping to the Disease Ontology and a subset of the UMLS*. The same 150 database instances used for the evaluation of textual snippet generation were also used to evaluate this task performed by Mapper.pl. For each of the 150 instances, a human reader selected the phenotype(s) most important to the captured age. These results were compared to those obtained by Mapper.pl.

### Hierarchal cluster analysis

For each Disease Ontology disease mapped to in the APK database, a count of the number of instances per age was obtained from the populated database. Evidence linked to inferred age ranges where excluded from this analysis. A matrix of disease-age instance count was generated and corrected for biases in literature coverage of diseases by dividing each disease-age count by the number of instances associated with that disease. Using Expander 5 software (Sharan et al. 2003), hierarchal clustering was conducted (similarity was measured using Pearson correlation).

### Availability

The APK database dumps file, the text mining scripts implemented in Perl, additional files and spreadsheets containing all the data regarding the evaluation of our text-mining pipeline can be found at: http://rubinlab.med.ad.bgu.ac.il/apk/APKSupplementary.html

The Age-Phenome data browser can be found at: http://rubinlab.med.ad.bgu.ac.il/apk/

The Age-Phenome Wiki can be found at: http://age-phenome-wiki.med.ad.bgu.ac.il/index.php?title=Main_Page

## Competing interests

The authors declare that they have no competing interests.

## Authors' contributions

NG and ER conceived the idea, designed the research and wrote the article; NG conducted the research. Both authors have read and approved the final manuscript.
